# Alexander Fleming: a second look

**DOI:** 10.5195/jmla.2024.1780

**Published:** 2024-01-16

**Authors:** Danielle Gerberi

**Affiliations:** 1 gerberi.danielle@mayo.edu, Supervisor, Saint Marys Staff Library and Rochester Patients' Libraries, Mayo Clinic Libraries, Rochester, MN

## Abstract

In 1928, Alexander Fleming (1881-1955) identified penicillin, the world's first antibiotic. It was a chance discovery that could have easily been missed had Fleming not taken a second look at a contaminated Petri dish. The discovery of penicillin marked a profound turning point in history as it was the first time deadly infections such as bacterial pneumonia, sepsis, diphtheria, meningitis, and puerperal fever after childbirth could be cured, and it paved the way for the development of additional antibiotics. The Alexander Fleming Laboratory Museum, one of several London Museums of Health and Medicine, is a reconstruction of Fleming's laboratory in its original location at St. Mary's Hospital. As if stepping back in time, visitors gain a glimpse into the man, his bacteriology work, and the events surrounding this important finding. For those unable to travel to London, this article provides a brief narrative of the fascinating story.

## INTRODUCTION

On a recent vacation in London, I happened upon the Alexander Fleming Laboratory Museum while checking Google Maps for points of interest near Paddington Station. For as much planning as I had done. reading multiple travel guidebooks and gathering recommendations on places to visit, I was completely unaware of this point of interest. Intrigued by the information I came across about Fleming through a quick online search, as well as the positive ratings on TripAdvisor.com, I readjusted my plans to include a stop at the museum located within Saint Mary's Hospital. While relatively small, it left a big impression and I highly recommend the museum to those visiting London who are interested in the history of medicine.

The Alexander Fleming Laboratory Museum is part of the collective London Museums of Health and Medicine which are sure to appeal to medical and health sciences archivists and librarians as well as to others seeking a little inspiration or perspective by looking to the past. Additional sites in this network include the Anaesthesia Heritage Centre, Barts Pathology Museum, British Dental Association Museum, Bethlem Museum of the Mind, British Red Cross Museum, Chelsea Physic Garden, College of Optometrists, Florence Nightingale Museum, Foundling Museum, Freud Museum, Hunterian Museum of the Royal College of Surgeons, Landon Down Museum, Museum of the Order of St. John, Old Operating Theatre and Herb Garret, Kew Royal Botanic Gardens, Royal College of Midwives, Royal College of Nursing Library and Heritage Centre, Royal College of Obstetricians and Gynaecologists, Royal College of Physicians Museum, Royal London Hospital Museum, Royal Pharmaceutical Society Museum, Royal Society of Medicine, Science Museum, St. Bartholomew's Hospital Museum, and St. George's Museum and Archives [1]. An entire trip to London could be spent visiting just a few of these gems!

An English Heritage blue plaque, one of almost a thousand across the city, marks the outside of the building at Saint Mary's Hospital as the location of the serendipitous discovery of penicillin in 1928. Opened in 1993 in the exact spot of the original lab and directed by archivist and Fleming biographer, Kevin Brown, the museum brings Fleming's paramount discovery to life.

**Figure 1:**
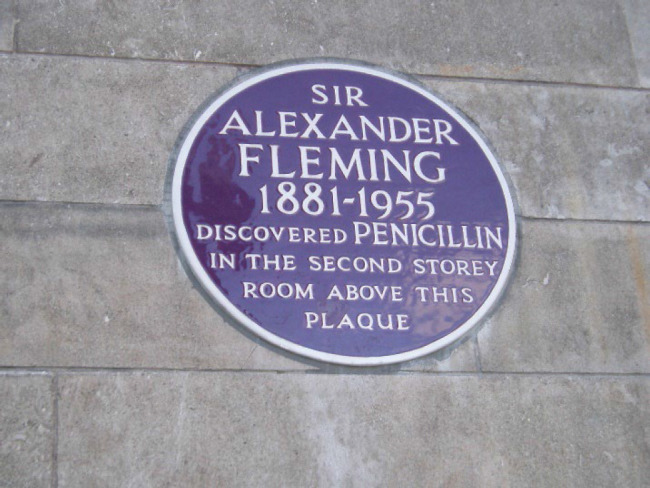
Alexander Fleming blue plaque by burge5k is licensed under CC BY 2.0

Alexander Fleming, known as Alec to his family, grew up on a farm south of Glasgow, Scotland. The second youngest of eight children, he cultivated the skill of keen observation when hunting and fishing as a boy roaming the countryside. After his father passed away at age seven, the family eventually moved to London. Advised as a teenager to pursue medicine as a rewarding profession by an older brother who went into ophthalmology, Fleming entered St. Mary's Medical School, the newest of the London teaching schools, in 1901. It is thought he arbitrarily applied to St. Mary's over other equidistant medical programs as he was aware of the comradeship of the water polo team; ironically, it was the rifle club in which he would become a valued teammate [2,11]. Fleming was a top student who also worked part-time as a research assistant in the Inoculation Department under the direction of Sir Almroth Wright, a pioneer of vaccine therapy who also became an influential mentor. Although Fleming planned on pursuing surgery and passed the Fellowship of the Royal College of Surgeons exam, he ultimately chose the specialty of laboratory medicine and would go on to spend his entire professional career practicing as a bacteriologist at St. Mary's Hospital.

**Figure 2:**
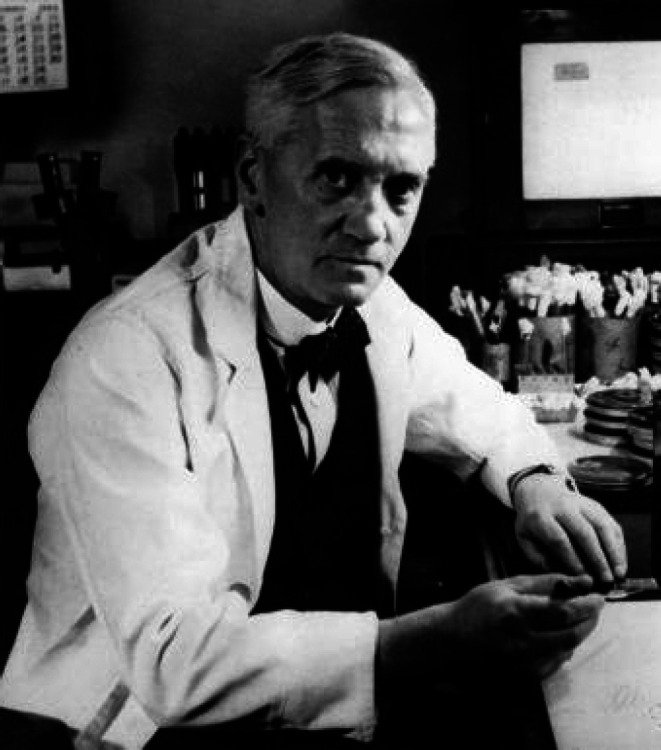
Picture of Alexander Fleming “File:Alexander Fleming3.jpg” by Calibuon at English Wikibooks, cropped by User:AlanM1 is marked with CC0 1.0.

At the onset of World War I, Wright was tasked with establishing a field laboratory at a base hospital in Boulogne, France to study projectile wound infections. Fleming accompanied Wright serving as a captain in the Royal Army Medical Corps. It soon became evident that explosive weaponry led to sepsis, gangrene, and tetanus causing large losses of life and limb. Fleming's research published in the Lancet in 1915 was the first extensive study of war wound infections. The team also demonstrated that the normative use of antiseptics, such as carbolic acid and sodium hypochlorite, damaged the body's protective leukocytes doing more harm than good. In both war and times of peace, Fleming strove to find a natural nontoxic bactericidal agent to treat infection [2].

In the winter of 1921, Fleming identified a substance in mucus and bodily fluids capable of fighting off bacteria and providing a level of natural immunity. He used a bad cold to his advantage to test his suspicions that nasal secretions are capable of lysis or cellular breakdown. After adding mucus to a culture plate and incubating for several weeks, the plate showed evidence of bacterial inhibition around the mucus. Further experiments using various samples from colleagues and animals resulted in consistent findings showing the trait was inherent and not due to a common cold infection. Wright proposed naming the active substance lysozyme since it acted similarly to an enzyme. When tested on pathogenic bacteria, lysozyme was ineffective and therefore unsuitable for practical therapeutic use, however, its discovery represented an advancement in the field of immunology [2, 6].

In 1928, Fleming was studying Staphylococcus in preparation to write a chapter on the topic for a bacteriology textbook. Before departing for vacation at the end of the summer, a mold spore, likely carried on clothing or through the air from the downstairs mycology lab, contaminated a Petri dish Fleming had cultured with Staphylococcus aureus. The culture plate was one of several left on the workbench for about a month to assess the effects of extended exposure to room temperature on the morphology and virulence of the bacterial colonies [2]. Upon returning to work, Fleming took a second look at a random culture plate that he intended to discard due to the mold contamination. Like his experience with lysozyme, he observed a clear, bacteria-free zone surrounding the mold growth. Under improbable environmental conditions of an unusually cool period of days followed by a heat wave during his vacation, “the contaminating mold had time to develop and the antibacterial agent it produced reached the colonies just at the right age and physiological state when they were still capable of dividing and, therefore, were susceptible to lysis under the influence of penicillin” [3]. Fleming realized the mold had secreted an antibacterial substance which he later named penicillin after the fungal genus Penicillium.

Fleming cultured the mold to evaluate its potency, bactericidal activity, interaction with leukocytes, as well as safety in animals. He found it to be nontoxic and effective against different Gram-positive bacteria such as streptococcus, pneumococcus, gonococcus, and meningococcus. He also conducted numerous experiments using different strains of Penicillium mold and other molds gathered from common sources. Remarkably, only one of the Penicillium strains obtained from the mycology lab exhibited the same effect [4]. Fleming published his landmark article, “On the bacterial action of cultures of a penicillium, with special reference to their use in the isolation of B. influenzae,” in the June 1929 issue of the British Journal of Experimental Pathology. Nonetheless, he and his assistants were challenged by the unstable nature of penicillin which made it difficult to extract and test in patients without a chemistry lab on hand. Fleming generously distributed mold samples around the world to other bacteriologists and mycologists for increased laboratory study but, despite working with penicillin throughout the 1930s as attested by his notebooks, no further breakthrough occurred until a decade later [2, 4, 5].

In 1939, biochemist Ernst Chain, a German-Jewish refugee, rediscovered Fleming's penicillin article while reviewing the literature on natural antibacterial substances. He had recently joined an interdisciplinary team at the Sir William Dunn School of Pathology at Oxford University directed by Australian pathologist Howard Florey. The Oxford team was interested in microbial antagonism since very little was known about the chemical or biological disposition of natural substances capable of inhibitory action on bacteria. Florey developed an interest in antibiosis after having read about Fleming's report on lysozyme in 1929 [2, 13]. The team decided to reexamine substances from past research including taking a fresh look at the antibiotic potential of penicillin. Coincidentally, a transfer from Fleming's preserved penicillin subculture was used. Chain exclaimed, “I was astounded at my luck in finding the very same mold about which I had been reading – here, and in the same building, right under our noses” [5].

Bolstered by chemistry expertise and equipment, the Oxford team developed a successful technique for isolating and purifying penicillin, however, it could only be generated in small quantities. After a controlled study in mice in 1940 showed promising therapeutic results, the notion of using penicillin as a life-saving drug in World War II became a driving force for accelerated human study. Early clinical use met with generally positive outcomes but was limited by the pressing need for a greater supply of penicillin [2, 17].

Interestingly, around this time, Fleming approached Florey to learn how the Oxford team was making penicillin and to ask for a purified dose for a critically ill patient at St. Mary's Hospital with streptococcal meningitis. Florey provided the medicine but asked Fleming to delay administering until he had tested as an intrathecal injection in an animal. Fleming ignored the request as the patient's condition was dire and there was little to lose. The next morning, Florey called Fleming to let him know the animal had died. Fleming revealed the patient's condition had vastly improved and the patient made a full recovery with continued treatment over the following weeks. The miraculous incident at St. Mary's Hospital and Fleming's name quickly spread through London media reports leading to celebrity status [2, 6].

Meanwhile, Florey sought a way to overcome the problem of inadequate penicillin supply using the existing surface culture method available in the lab. Pharmaceutical companies in the United Kingdom were stretched for resources supporting the war effort so Florey headed across the Atlantic to seek outlets for industrial production. Once in the United States, a referral was made to the US Department of Agriculture's Northern Regional Research Laboratory in Peoria, Illinois, which operated a newly developed fermentation facility in the heart of the corn belt. Submerged fermentation, involving aeration of a liquid byproduct from corn in large stainless-steel tanks, proved to be a much more efficient method for growing mold. Combined with a fluke finding of an extra productive strain of mold on a locally grown cantaloupe melon, an exponential yield could be achieved [2, 4].

Coinciding with the American entry into World War II, two federal government agencies, the Office of Scientific Research and Development and the War Production Board, effectively mobilized resources driven by the urgent medical defense needs of the armed forces. A major aim was to generate a sufficient supply of penicillin in preparation for the D-Day invasion on June 6, 1944, since the ability to ward off infection might mean the difference between triumph and defeat. A monumental cooperative effort ensued involving the government, academic institutions, research foundations, and over twenty pharmaceutical companies. Unprecedented free exchange of technical information rapidly accelerated manufacturing. Across the pond, Fleming and Florey advocated scaled-up production in the UK which was hampered by shortages of workers and raw materials, not to mention air raids. Progress was eventually achieved to adequately treat wounded allied troops on multiple war fronts [2, 14, 15].

With more penicillin on hand, Florey was able to move ahead with clinical trials at home as well as on the front lines in North Africa which met with great success. In a trial initiated at St. Mary's Hospital, Fleming was insistent that patients first be tested for diagnostic sensitivity to penicillin before administering the drug since he knew penicillin was not a cure-all. Trials in the US began in 1943 on wounded soldiers at hospitals in Utah and New York with conclusively positive results [2].

Pharmaceutical advertisements displayed slogans such as, “Thanks to Penicillin…He Will Come Home!” serving as some reassurance to families on the home front [8]. Word spread on the life-saving power of penicillin and, for a time, “it became the most sought-after commodity in the world” [9]. Demand escalated for the precious drug for acutely ill civilian cases but penicillin was initially strictly reserved for military use. By 1946, the so-called ‘wonder drug' was finally available for the public. As production increased, the price dropped from nearly priceless in 1940, to $20 per dose in July 1943, to $0.55 per dose three years later [10].

**Figure 3:**
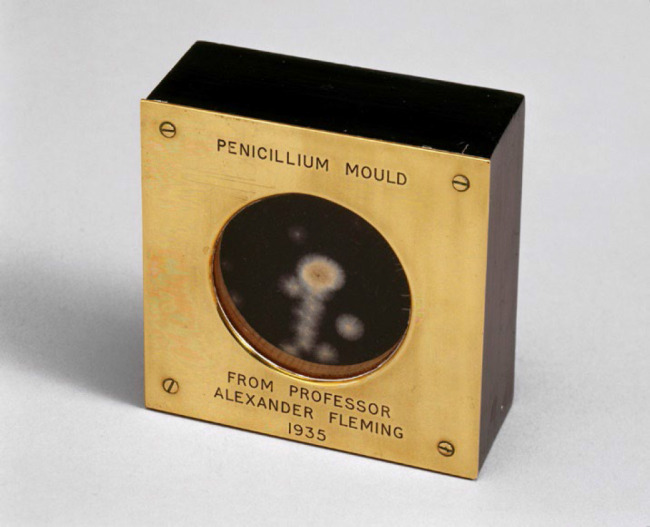
Example of one of Fleming's ‘mold medallions' from Science Museum London / Science and Society Picture Library; licensed under CC BY-SA 2.0.

Outside of North America and the UK, global demand arose for penicillin despite countries such as France, the Netherlands, China, Japan, and Germany attempting to manufacture it independently during the war. Reportedly, Hitler was treated with penicillin after the July 1944 inside failed assassination attempt, yet Germany was slow to realize mass production of penicillin. “Although some German scientists and administrators did recognize the importance of penicillin, their efforts were frustrated by infighting, greed and poor organization” in contrast to the coordinated program in the U.S. [17, 18]. The United Nations, World Health Organization and U.S. and Canadian governments assisted with the post-war reconstruction efforts to supply Penicillium cultures, equipment, funding, and training to European and Asian countries such as Italy, Poland, India, and Japan [2, 16].

For their critical involvement in the discovery of penicillin, Fleming, Florey, and Chain were jointly awarded the Nobel Prize in 1945. Dorothy Crowfoot Hodgkin, a chemist at Oxford, determined the molecular structure of penicillin through x-ray crystallography that same year and she was later awarded the Nobel Prize for chemistry in 1964. Chemist John Sheehan successfully created synthetic penicillin in 1957 after nine years of research at the Massachusetts Institute of Technology, though researchers were quick to develop new antibiotics for bacterial infections unresponsive to penicillin.

Fleming was knighted for his service to humanity and granted numerous honors. Somewhat at odds with his unassuming personality, Fleming traveled extensively overseas in a public ambassador role to worldwide acclaim. He presented handcrafted ‘mold medallions' as gifts which he made by sealing mold grown on blotting paper between spare eyeglass lenses from his brother's ophthalmology practice. Notable recipients of the mold medallions included the Queen of England, “Churchill and Roosevelt, friends, colleagues, and many of the people he met on his numerous travels. These insignificant-looking artifacts soon took on the status of holy relics” [2].

Although the discovery of penicillin has been called a fortuitous accident by some, Fleming's acumen certainly played a role. Louis Pasteur's statement, “chance only favors the mind which is prepared,” from an 1854 opening address has been evoked in describing Fleming's reaction on the fateful day he encountered the contaminated Petri dish and gave it a second look [2, 6, 7]. Unquestionably, Fleming succeeded in his quest to find a safe substance capable of destroying lethal bacteria which consequently saved countless lives.

Lesser known interesting facts about Fleming include he was called “Flem” by his colleagues, he almost always wore a colorful bow tie, he had an artistic bent which extended to the lab where he created miniature detailed paintings and gardens with pigment-producing microbes, he was an exceptional glass blower who custom-made laboratory apparatus for his own experiments, he conducted the first documented systematic study of nosocomial infection, he introduced a black staining dye for bacteria known as nigrosin, he was fortunate to be unharmed when his London home was hit by bombs in 1941, he perceptively expressed concerns about penicillin overuse leading to antibiotic resistance, his ashes are interred at St Paul's Cathedral alongside tombs and memorials of national British heroes such as Admiral Lord Nelson, the Duke of Wellington and Florence Nightingale, his notebooks are held by the British Library, and his original penicillium mold plate resides at the British Museum [6, 9,11].

Fleming's apprehension about antibiotic resistance proved all too true. Tragically, as the one-hundredth anniversary of the discovery of penicillin draws near, the efficacy of the once remarkable “wonder drug” and successive antibiotics has greatly diminished with the rise of antimicrobial resistance from widespread indiscriminate use. According to the World Health Organization, antimicrobial resistance is one of the top 10 global public health threats facing humanity [19]. Combating this serious threat requires collaborative committed action from intergovernmental bodies, the healthcare and agriculture sectors, academia as well as the public. Emerging alternative antimicrobial approaches, such as nanoparticles and phagotherapy, along with drug repurposing guided by artificial intelligence, are a few strategies offering some hope in a post-antibiotic era [20].

Visiting the Alexander Fleming Laboratory Museum prompted several reflections. Publication and literature searching, so important to our professional work, were influential at multiple stages in the turn of events. The saying, “there is always more to the story” could not be more fitting as many unnamed contributions were made along the way. “An interdisciplinary approach was needed for the development of penicillin. No single investigator, or small group of investigators, could have accomplished this task” [10]. Even Illinois farms were acknowledged in an online reference I came across which caused me to wonder if my maternal grandmother's family from a farm in central Illinois could have played a tiny part supporting the fermentation process at the Northern Regional Research Laboratory in Peoria [12]. The momentum of World War II facilitated exceptional public-private partnerships to make penicillin a timely reality, changing the course of history. My paternal grandfather, Michael Gerberi, who survived landing on Omaha Beach in the invasion of Normandy on D-Day but was later injured in the Battle of the Bulge and developed pneumonia, may have owed his life in part to penicillin. Perhaps a practical key takeaway is that we can do ourselves and others a service by making a point to take a second look.
